# Heart rate of fire: exploring direct implementation of physiological measurements in realistic shoot/don't-shoot simulations

**DOI:** 10.3389/fspor.2024.1444655

**Published:** 2024-08-29

**Authors:** Adam T. Biggs, Andrew E. Jensen, Karen R. Kelly

**Affiliations:** ^1^ Medical Department, Naval Special Warfare Command, San Diego, CA, United States; ^2^ Leidos, Inc., San Diego, CA, United States; ^3^ Warfighter Performance Department, Naval Health Research Center, San Diego, CA, United States

**Keywords:** shooting, simulator, rate of fire, heart rate variability, semi-automatic, automatic

## Abstract

**Introduction:**

Shooting simulations provide an excellent opportunity to train use-of-force decisions in controlled environments. Recently, military and law enforcement organizations have expressed a growing desire to integrate physiological measurement into simulations for training and feedback purposes. Although participants can easily wear physiological monitors in these scenarios, direct implementation into training may not be simple. Theoretical problems exist in the ultra-short heart rate variability windows associated with use-of-force training, and practical problems emerge as existing scenario libraries at training organizations were not designed for physiological monitoring.

**Methods:**

The current study explored the challenges and possibilities associated with direct implementation of physiological monitoring into an existing library of firearms training scenarios. Participants completed scenarios in a shooting simulator using existing military training scenarios while wearing a device to monitor their heart rate.

**Results:**

The results revealed lower heart rate variability (approximately 6%) occurred in scenarios where participants did not have to fire weapons, indicating that don't-shoot scenarios may actually impose more cognitive stress on shooters. Additional evidence further demonstrated how both behavioral and physiological factors could be used concomitantly to predict unintentionally firing on non-hostile actors. However, behavioral measures were more predictive (e.g., *β* = .221) than physiological measures (e.g., *β* = −.132) when the latter metrics were limited to specific scenarios. Qualitative results suggest that simply applying physiological monitoring to existing shooting simulations may not yield optimal results because it would be difficult to directly integrate physiological measurement in a meaningful way without re-designing some elements of the simulations, the training procedure, or both.

**Discussion:**

Future use-of-force shooting simulations should consider designing novel scenarios around the physiological measurement rather than directly implementing physiological assessments into existing libraries of scenarios.

## Introduction

Use-of-force encounters denote any scenarios wherein physical intervention becomes required to diffuse a situation. This label can include a continuum of non-lethal and lethal means—including handcuffs, tasers, and firearms—which each introduce complexity about the challenges of their use and the stress imposed during the encounter (1). The threat inherent to use-of-force encounters often imposes significant stress upon the individuals involved through a complex interaction of physiological and cognitive factors. Moreover, preparing military personnel, law enforcement, and other individuals to use force requires intense training and appropriate identification of field readiness. Corresponding training programs in turn attempt to use a variety of methods to safely train and develop use-of-force skills among personnel before certifying them for field readiness. Indeed, there are so many military and law enforcement training programs that the existing efforts far outnumber the research efforts supporting innovation in this training. Still, shrewd trainers regularly seek to augment their existing programs with the best science and technology available. Given the substantial evolution of wearable technology (2), and the physiological stress involved during use-of force encounters, there has been a growing desire to integrate off-the-shelf commercial wearable technology into shooting simulations during use-of-force training. The idea appears sound—trainers can gain insight into stress responses among trainees. Nevertheless, direct implementation raises a critical question: can physiological monitoring be implanted off-the-shelf into use-of-force training programs?

Recent evidence has demonstrated that physiological differences can predict shooting performance (3), but marksmanship is not the sole source of error in a use-of-force simulation (4). Stress may induce cognitive failures that predispose the individual to errors such as firing on an unarmed person (5). Additionally, physiological monitoring offers the potential for a single instructor to gain deeper insight into the reactions of a larger training group. Consider a single instructor evaluating a class of students before they engage in live-fire exercises, where physiological monitoring could augment their situational awareness and help identify students who might be overstressed by a particular training procedure—allowing them to intervene before excessive stress becomes a potentially fatal training outcome. This fundamental concept is similar to athletic performance in which physiological shifts can predict individual performance (6–11). While validated in sport, integration into predicting errant use-of-force outcomes for military or law enforcement has been more limited in scope [cf. (3, 12)]. Thus, the current effort aims to address this gap through integration of physiological monitors into pre-existing shooting simulations rather than new scenarios created for experimental purposes.

Currently, military and law enforcement personnel routinely simulate stress under controlled, scenario-based conditions to prepare personnel for use-of-force encounters (13–18). Through these efforts, evidence indicates that anxious officers are more inclined to shoot (19, 20), which represents one complex physiological and cognitive factor that can predispose individuals to shooting errors. This challenge is particularly problematic given that practice alone does not overcome some biases and prevent errors (21)—a conclusion comparable to other indications that shooting qualifications alone may not adequately predict high-stress field shooting performance (22).

One prominent training method involves the use of shooting simulators to provide controlled experience in making shoot-don't shoot decisions. Traditional marksmanship training typically utilizes static or predictable elements such as unmoving targets at a known distance with a start signal. Alternatively, simulator training can present a shooter with dynamic scenarios of changing variables that unfold over time while intermixing hostile and non-hostile actors. These simulations produce a more realistic shoot/don't-shoot decision since the choice to engage a target comes from the shooter rather than a start signal. Simulators have already been vetted against live fire marksmanship on a static flat range (e.g., a marksmanship range where shooters remain behind a shooting line and do not move) as a viable proxy for training because simulator marksmanship can predict performance with live rounds (23–25). Further, simulators excel at creating a wide variety of immersive scenarios in a way that would not be practical within a force-on-force training (e.g., role players, rather than paper targets, use non-lethal surrogates to simulate a use-of-force encounter) exercise given safety and facility limitations [cf. (26)]. Recent research has explored numerous facets about the psychophysical relationship of shooting decisions that might benefit these simulations, including the immersive experience in virtual reality (27), death anxiety in a simulated shooting engagement (28), and how introducing a pain stimulus such as shock can increase anxiety in virtual reality (19, 29).

Among military and law enforcement training personnel, there is a strong desire to treat physiological monitoring as a plug-and-play-style measurement tool into existing libraries of shooting simulations. This desire stems from practicality as there are more shooting simulators across military and law enforcement organizations than there are physiologists to properly staff them. Meanwhile, physiological monitoring devices have become exceptionally common with growing and widespread use (30, 31). The combination of widespread adoption, stress monitoring capability, and commercial availability makes physiological monitoring a growing area of interest among use-of-force training. However, existing training programs were not designed for physiological monitoring. For example, shooting simulators often have existing libraries of pre-programmed scenarios designed to mimic the intensity and brevity of a shooting engagement without concern for the requirements of valid physiological monitoring, such as sufficient baseline for measurement or washout periods between scenarios. This limitation creates theoretical problems associated with the validity of ultra-short heart rate variability (HRV) metrics (32–36); but see also (37), and practical problems associated with interpreting results between scenarios given how the technology measures physiological characteristics. Unfortunately, despite these theoretical and practical limitations, there is a distinct probability that the applied solution will be the plug-and-play approach to integrating physiological monitoring into shooting simulations. In turn, there is a need to understand the practical consequences of this implementation.

The current study is a practical exploration for direct implementation of physiological monitoring into existing libraries of training scenarios for military, law enforcement, and security training organizations. There were two specific goals to this effort. The first goal was to provide lessons learned from a direct integration of physiological monitoring into existing libraries of shooting simulations. These lessons learned could serve as a basis for organizations considering direct implementation of physiological monitoring into shooting simulations. The second goal was to measure any relationship between physiological variables, scenario types, and the weapon rate of fire (automatic vs. semi-automatic). Specifically, automatic weapons create the potential for substantial unintended casualties and collateral damage given that each additional round fired represents another opportunity for either an accurate shot or a misfire that strikes an unintended target (38). These implications may vary substantially across scenario types, which are broadly divided into 3 categories here: (1) don't-shoot scenarios, where participants should never fire the weapon because they never encounter a hostile actor; (2) shoot scenarios, where the participant should fire the weapon, but may encounter a mix of hostile and non-hostile actors; (3) fluid scenarios, where actors may present as hostile or non-hostile initially yet change their presentation during the scenario (e.g., present initially as a non-hostile, but then draw a weapon and begin firing). Fluid hostility scenarios in particular create the potential for dynamic intervention as the participant must maintain high situational awareness and monitor all aspects of a scenario. These combined elements thus present a realistic decision-making environment for the shooting simulations. To satisfy the ecological validity requirement, this investigation utilized the United States Marine Corps (USMC) indoor Simulated Marksmanship Trainer (ISMT). This platform has been regularly used for decades by USMC training programs to prepare personnel for potential use-of-force scenarios by exposing them to use-of-force situations in a controlled environment.

## Methods

### Participants

Eighteen adults (age: *M *= 31.33, *SD *= 4.67; 12 males, 6 females) were included in the study as volunteers with normal or corrected-to-normal vision. All participants were recruited from the community sample around Wright-Patterson Air Force Base. A total of 25 individuals initially participated in the experiment. However, physiological data were not collected for one participant, and a computer malfunction resulted in the loss of behavioral shooting data for six participants.

The Naval Medical Research Unit Dayton Institutional Review Board approved the study protocol (NAMRUD.2017.2011) and oversaw compliance with all applicable federal regulations governing the protection of human participants. All participants gave voluntary consent to participate.

### Shooting simulator

**Figure 1:**
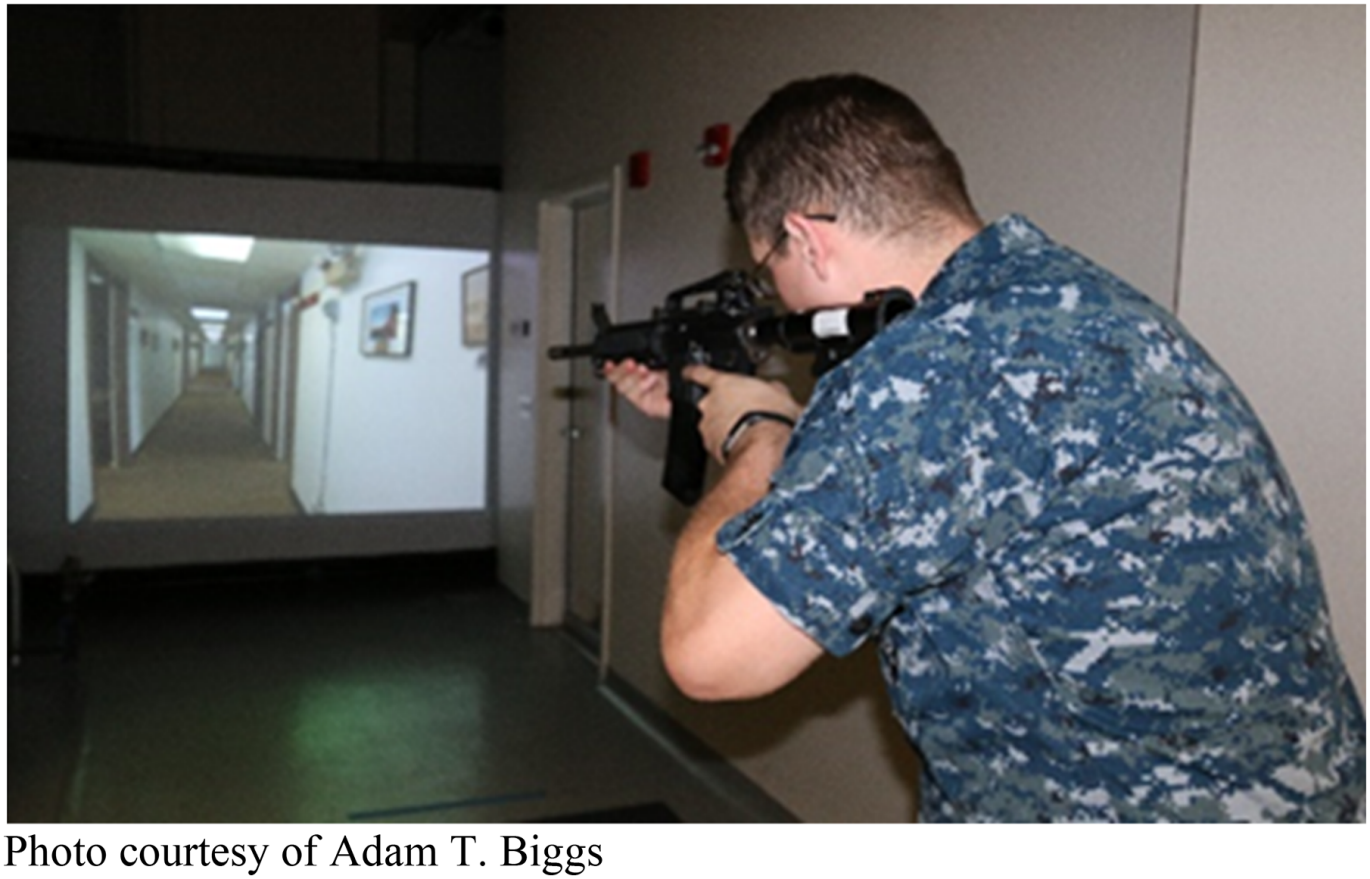
Sample image of a shooter participating in an indoor simulated marksmanship trainer simulation.

Shooting scenarios were conducted using the Indoor Simulated Marksmanship Trainer (ISMT; Meggitt Training Systems, Suwanee, GA) (see Figure 1). This simulator system is the primary USMC training simulator. Each simulator included an operator computer station connected to an infrared hit camera and projector, which displays the image onto a screen while detecting where the infrared laser struck the screen to determine hits and misses. The ISMT projects an image onto a screen (75-in width × 57-in height) while participants stand a standardized distance (15 feet) from the screen (shooting line).

Simulated weapons were Bluefire® weaponry (Meggitt), which replace inner components of a functioning weapon with an infrared laser and Bluetooth transmitter. Magazines contained compressed air and simulated an actionable recoil while speakers emitted a sound to simulate gun fire. All participants used an M4 rifle for all shooting tasks with iron sights and no enhanced optics. All rifles were zeroed and confirmed by an experimenter prior to each experimental session. The rifle selector switch was set to “semi” and fired one round for each trigger pull for semi-automatic (SEMI) condition. In the automatic condition (AUTO), the selector switch was set to “auto”, with a 700–950 round/min cyclic rate of fire. During AUTO conditions, a trigger pull would initiate the continuous firing of rounds until the participant released the trigger or the weapon ran out of “ammunition”. Ammunition was limited by compressed air in the magazine. Note that the magazines are designed to hold up to 30 shots worth of compressed air, although extended use can wear out the cartridges and make it possible that a particular cartridge might fire less (e.g., O-rings lose their snug fit from repeated use). Air cartridges were checked regularly throughout the study and removed if identified that their cartridges could not hold the full 30 rounds.

Familiarization with the ISMT was achieved as has been previously described [see (4)]. Participants were instructed to fire upon hostile targets as they presented and to avoid shooting at non-hostiles during the scenario. Performance was determined from video replay following the experimental session by a member of the research team and validation was conducted by a second member of the research team. Feedback was indicated for each shot based on the color of the circle where the shot landed: green indicated a complete miss of either hostile or non-hostile, yellow indicated a non-lethal hit on a hostile target, red indicated a lethal hit on a hostile target, and maroon indicated a shot striking a non-hostile, regardless of where the round struck the actor. The ISMT provides the feedback as part of the scenario programming. First shot reaction time (RT) was identified as the time between presentation of the first hostile threat and the first round fired within a given scenario. For example, if a hostile target appeared 10.00 s into the scenario and time to the first shot of target engagement was 11.35 s, the first shot RT would be recorded as 1.35 s.

### Shooting scenarios

Participants engaged in 32 unique simulated video scenarios across both the SEMI and AUTO weapons conditions. Four different presentation orders were created to counterbalance the order for weapon rate of fire and unique scenario order. Two unique sets of 16 videos were created to equate the number of hostile actors appearing during the block. Half of the participants were assigned to complete video set A with a weapon on SEMI rate of fire, and the other half were assigned to complete video set B with a weapon on AUTO rate of fire. Video sets A and B were also counterbalanced as to which order the participant completed first. Participants completed both halves during the same experimental session. This counterbalancing scheme created four different potential orders to which participants were randomly assigned. There were 29 total hostile actors across the 32 unique scenarios (7 scenarios with 0 hostiles; 21 scenarios with 1 hostile; 4 scenarios with 2 hostiles), and 37 non-hostiles across the 32 unique scenarios (8 scenarios with 0 non-hostiles; 15 scenarios with 1 non-hostile; 7 scenarios with 2 non-hostiles; 1 scenario with 3 non-hostiles; and 1 scenario with 5 non-hostiles). The high proportion of hostile actors across the scenarios was intentional to produce a strong prepotent motor response (39, 40).

These scenarios were broadly categorized as don't-shoot scenarios (*N* = 7), shoot scenarios (*N* = 21), and fluid hostility scenarios (*N* = 4). Don't-shoot scenarios included only non-hostile actors who never presented a threat, and shoot scenarios included hostile actors who presented as immediate threats. Hostility scenarios included hostile actors who initially presented as non-hostile and then revealed themselves as a threat. The goal was to keep an approximately 80%–20% ratio of go-to-no-go that would ensure a strong prepotent motor response (39). A go trial was any scenario where the shooter should have fired the weapon at some point, or 25/32 scenarios (78.13%).

Equipment malfunctions were possible during the shooting scenarios due to either a mechanical error with the firearm (e.g., weapon jammed and the participant had to clear the jam) or human error (e.g., participant left the safety on). In all cases, the shooting scenarios were only included in data analyses if there was no mechanical error or human error. There were 14 instances (2.43%) of weapon malfunction due to mechanical error (e.g., gun jammed), 8 instances (1.39%) of weapon malfunction due to human error (e.g., safety left on), and 12 instances (2.08%) of computer malfunction. These limitations amounted to a loss of 5.90% of data. Mechanical and human errors were not included with the current analyses because the focus here involved physiological reactions while using the weapons. Errors, such as a mechanical failure, weapon jam, or leaving the safety on, did not occur often enough for any robust statistical analyses. For example, gun jams did occur, albeit infrequently and across different scenarios that prevented accumulating enough data for analysis in any particular situation. The final behavioral dataset included 541 shooting scenarios across 18 participants. Regression analyses were used for all scenarios, whereas analysis of variance (ANOVA) tests were used only the behavioral data at the participant level (i.e., averaged across scenarios).

### Physiological monitoring

All participants wore a Zephyr™ BioHarness™ system (Medtronic, Boulder, CO) throughout the shooting scenarios. Prior to engaging in experimental scenarios, resting baseline HR was measured for 5 min while participants stood. During the experimental scenarios, HRV was measured by the standard deviation of normal-to-normal (SDNN) intervals in milliseconds. As per the technical manual [for full details, see (41)], the device calculates HRV (i.e., SDNN) in the time domain (i.e., milliseconds) by using a rolling 300-beat SDNN value. No data is captured for the first 300 heartbeats (approximately 5 min) to create this rolling average, and afterward, the reporting frequency is collected at 1 Hz thereafter to provide a current value. The rolling average of SDNN reduces random artifact in reporting of HRV values. The average scenario was completed in 43 s, or an average of 11 min and 28 s per block. Notably, there was no washout period between scenarios. Participants completed one scenario before immediately moving onto the next. This procedure better resembles how shooting simulator use occurs in training rather than how experimental physiology studies might partition trials for data separation.

### Data and statistical analyses

Data analyses were conducted using IBM® SPSS® software (Armonk, NY). All raw values are presented as means or mean differences ± standard error, mean (±SE). For any missing values when conducting analyses of variance (ANOVAs), missing values were replaced with the group mean prior to running analyses. This method only applied to 1 participant whose behavioral shooting data in the fluid hostility scenario was lost. Multiple *post hoc* comparisons were corrected using the Bonferroni method.

A repeated measures multivariate ANOVA (MANOVA) was conducted to determine whether there were behavioral differences in shooting performance between the scenario type (shoot or fluid hostility) and weapon rate of fire (SEMI or AUTO). Five dependent variables were included: number of shots fired, number of lethal rounds fired, number of non-lethal rounds fired, number of false alarms (unintended casualties), and first shot RT. Because the behavioral variables were largely exploring the consequence of engaging the target, the scenario type was limited only to the shoot and fluid hostility with the don't-shoot scenarios excluded from these MANOVA analyses. Specifically, excellent performance in a don't-shoot scenario would involve not firing any shots, and 4 of the 5 dependent variables (shots fired, lethal rounds fired, non-lethal rounds fired, and first shot RT) would all describe errors alone since successful performance would yield no data. False alarms for don't-shoot scenarios were thus analyzed separately due to the behavioral implications.

To predict unintended casualties inflicted during the scenario, also described as false alarms, logistic regression was used in the analyses to determine whether a false alarm occurred during a given simulation. This approach recoded false alarms as a binary variable (present or absent) with any scenario including multiple false alarms counted as having a false alarm present. Behavioral and scenario variables included total shots fired during the scenario, number of non-hostiles in the scene, weapon rate of fire (categorical: SEMI or AUTO), and number of lethal rounds fired in the scenario. Physiological variables included HRV, %HR, and age. First shot RT was not included as a predictor variable because many don't-shoot scenario trials with an unintended casualty had missing data specifically for first shot RT (an experimenter recorded only the false alarm, not the time). These analyses were limited only to scenarios with at least 1 non-hostile present to create the potential for this error, and analyses were collapsed across all participants, meaning that the data were analyzed at the trial level. These limitations left 297 total scenarios in the data with 74 scenarios (24.92%) including a shot fired at a non-hostile and 223 scenarios (75.08%) with no shots fired at non-hostiles. A second logistic regression was conducted without requiring the shots fired criterion, which left 409 total scenarios in the dataset with 74 scenarios that included a shot fired at a non-hostile. As such, the 74 instances of firing upon non-hostile targets satisfied the logistic regression criterion of at least 5–9 outcome events per predictor variable (42).

To predict accurate performance during shooting scenarios, a linear regression and multiple variables were used to predict the number of lethal rounds fired. Behavioral and scenario predictors included total shots fired during the scenario, first shot RT, number of non-hostiles in the scene, and weapon rate of fire (categorical: SEMI or AUTO). Physiological variables included HRV, percentage of maximum heart rate (%HR), and age. The potential for collinearity issues was first assessed given variables such as shots fired and first shot RT. No predictor variable had a variance inflation factor above 1.52, indicating that collinearity was not an issue for this set of predictors (43). This analysis was performed with trial-level data and limited to scenarios with at least one shot fired to ensure that a lethal shot was a possible outcome. These limitations left 381 different scenarios across all participants for analysis.

## Results

### Behavioral analyses

Shooting behaviors were quantitatively measured as a methodological check to ensure performance adhered to expectations. Specifically, if participants are behaving as expected in a shooting simulation, then weapon rate of fire should significantly alter their behavior. This information is thus presented as a methodological check on behavior and for practitioner interest given possible differences on accuracy in shooting behaviors due to scenarios and weapon type. A 2 × 2 repeated measures MANOVA was conducted to determine whether there were behavioral differences between the different shooting scenarios. Within-subjects factors included scenario (shoot, fluid hostility) and weapon rate of fire (SEMI or AUTO), with the dependent variables of shots fired, lethal hits, non-lethal hits, false alarms, and first shot RT. Behavioral data from don't-shoot scenarios are analyzed separately within this section for reasons outlined in the Methods. Results are presented in Table 1.

**Table 1:** Descriptive statistics for shooting behaviors, divided by scenario type and weapon rate of fire.

Dependent variable	Shoot scenarios		Fluid hostility scenarios	
	AUTO	SEMI	AUTO	SEMI
Shots fired^a^	9.14 (0.89)	4.35 (0.58)	8.24 (1.06)	4.97 (1.11)
Lethal hits^a^	1.55 (0.18)	0.96 (0.14)	2.32 (0.50)	1.31 (0.19)
Non-lethal hits^a^	1.95 (0.21)	1.15 (0.17)	2.35 (0.37)	1.61 (0.31)
False alarms^b^	0.16 (0.05)	0.15 (0.03)	0.06 (0.06)	0.06 (0.04)
First shot RT^c^	2.00 (0.23)	1.81 (0.13)	1.46 (0.12)	1.49 (0.09)

Data are presented as mean (± SE). RT, response time; AUTO, automatic rate of fire; SEMI, semi-automatic rate of fire.

^a^ Described in rounds/scenario.

^b^ Described as false alarms/scenario.

^c^ Described as the time delay (in seconds) between the actor presenting a hostile threat and the first fired shot.

There was a significant omnibus effect in the multivariate analysis for scenario type, Wilks' Λ = 0.21, *F*(5, 13) = 9.91, *p* < .001, *η*_p_^2^ = .79, and for weapon rate of fire, Wilks' Λ = 0.17, *F*(5, 13) = 12.82, *p* < .001, *η*_p_^2^ = .83. The interaction was not significant, Wilks' Λ = 0.76, *F*(5, 13) = 0.81, *p* = .56, *η*_p_^2^ = .24. Univariate tests and *post hoc* comparisons were conducted to explore the differences. For scenario-based differences, there were significant univariate effects of lethal rounds fired, *F*(1, 17) = 7.67, *p* = .01, *η*_p_^2^ = .31; false alarms, *F*(1, 17) = 21.78, *p* < .001, *η*_p_^2^ = .56; and first shot RT, *F*(1, 17) = 15.12, *p* < .01, *η*_p_^2^ = .47. There was no main effect of shots fired, *F*(1, 17) = 0.14, *p* = .72, *η*_p_^2^ < .01, or of non-lethal rounds fired, *F*(1, 17) = 3.72, *p* = .07, *η*_p_^2^ = .18. Post hoc comparisons supported a performance advantage for participants in a fluid hostility scenario. This advantage was observed as more lethal rounds fired [0.56 rounds/scenario ±0.20 rounds/scenario, 95% confidence interval (CI) (0.13, 0.98), *p* = .01, *d* = 0.57], fewer false alarms [−0.09 false alarms/scenario ± 0.02 false alarms/scenario, 95% CI (−0.14, −0.05), *p* < .01, *d* = 0.64], and a faster first shot RT [−0.43s ± 0.11s, 95% CI (−0.66, −0.20), *p* < .01, *d* = 1.07]. Participants fired 45% more lethal rounds in fluid hostility scenarios and were nearly a half second faster on the first shot.

**Figure 2:**
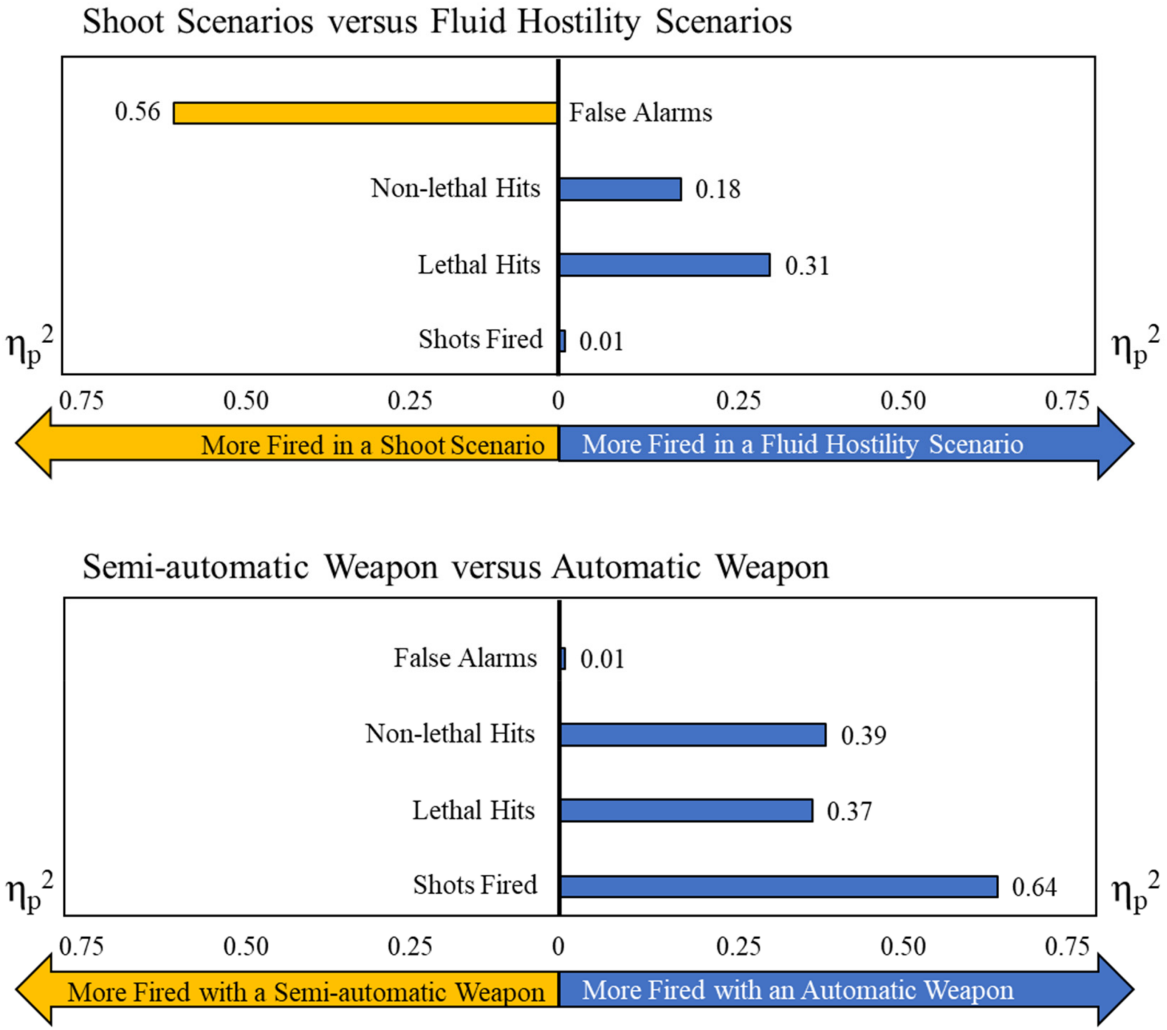
Effect sizes (*η*_p_^2^) as measured between the scenarios (top) and between weapon rates of fire (bottom). The measurement is depicted on the side with more instances of the event. For example, the top image depicts false alarms on the left side to represent that more false alarms were fired in a shoot scenario than in a fluid hostility scenario (*η*_p_^2^ = 0.56).

For weapon rate of fire differences, there were significant univariate effects of shots fired, *F*(1, 17) = 30.46, *p* < .001, *η*_p_^2^ = .64; lethal rounds fired, *F*(1, 17) = 9.85, *p* < .01, *η*_p_^2^ = .37; and non-lethal rounds fired, *F*(1, 17) = 10.96, *p* < .01, *η*_p_^2^ = .39. There was no main effect of false alarms, *F*(1, 17) = 0.03, *p* = .88, *η*_p_^2^ < .01, and no main effect of first shot RT, *F*(1, 17) = 0.14, *p* = .72, *η*_p_^2^ < .01. Post hoc comparisons supported a performance advantage for participants with an AUTO weapon. This advantage was observed as more shots fired [4.02 rounds/scenario ± 0.73 rounds/scenario, 95% CI (2.49, 5.56), *p* < .001, *d* = 1.24], more lethal rounds fired [0.80 rounds/scenario ± 0.26 rounds/scenario, 95% CI (0.26, 1.35), *p* < .01, *d* = 0.78], and more non-lethal rounds fired [0.77 rounds/scenario ± 0.23 rounds/scenario, 95% CI (0.28, 1.26), *p* < .01, *d* = 0.81]. In short, participants fired 86% more shots per scenario with an AUTO weapon, which may have also driven the increased number of lethal (71%) and non-lethal rounds (56%) fired during the scenario (see Figure 2).

### Accuracy

There was no significant effect of weapon rate of fire (SEMI or AUTO) on accuracy in this analysis, *Χ*^2^(3, *N* = 406) = 4.47, *p *= .22. This analysis was limited to the shoot and fluid hostility scenarios.

### False alarm

Although most performance metrics could not include the don't-shoot scenario, the false alarm analysis must be addressed separately to include these simulations. A 3 × 2 chi-square analysis was conducted on all scenarios, with the first factor representing the scenario type (don't-shoot, shoot, fluid hostility) and the second factor representing whether a false alarm occurred (present or absent). This analysis was limited only to scenarios with at least 1 non-hostile, to allow for the potential of a false alarm. There was a significant effect of scenario type on the likelihood of committing a false alarm, χ^2^(2, *N* = 410) = 8.17, *p *= .02. False alarm rates were comparable for shoot scenarios (20.28%; 44/173) and don't-shoot scenarios (20.80%; 26/125), but lower in fluid hostility scenarios (5.88%; 4/68).

### Physiological analyses

When averaging across all scenarios and weapon rates of fire, there was a significant increase in HR over baseline [mean difference = 13.15 bpm, SE = 2.81 bpm; *t*(23) = 4.69, *p *< .001, Cohen's *d *= 1.11]. A 3 × 2 repeated measures MANOVA was conducted to determine whether there were differences in the physiological stress imposed by the different shooting scenarios. Within-subjects factors included scenario (don't-shoot, shoot, fluid hostility) and weapon rate of fire (SEMI or AUTO), with the dependent variables of HRV and peak HR (in bpm) during the scenario. See Table 2 for results and Table 3 for an omnibus comparison to behavioral analyses.

**Table 2:** Descriptive statistics for physiological measures, divided by scenario type and weapon rate of fire.

Dependent variable	Don't- shoot scenarios		Shoot scenarios		Fluid hostility scenarios	
	AUTO	SEMI	AUTO	SEMI	AUTO	SEMI
Peak HR^a^	100.37 (2.92)	101.22 (2.84)	99.79 (2.69)	100.92 (2.73)	100.14 (2.78)	101.49 (2.81)
Average HR^a^	91.92 (2.88)	93.01 (2.80)	90.49 (2.80)	92.78 (2.81)	91.35 (2.76)	92.84 (2.81)
Average HRV^b^	51.05 (3.49)	49.35 (4.03)	54.46 (4.17)	52.95 (3.79)	54.58 (4.09)	51.64 (3.98)
Max HR %^c^	53.17% (1.58%)	53.61% (1.53%)	52.86% (1.45%)	53.47% (1.50%)	53.05% (1.50%)	53.75% (1.50%)

Data are presented as mean (± SE). HR, heart rate; AUTO, automatic rate of fire; SEMI, semi-automatic rate of fire.

^a^ Described in beats per minute.

^b^ Described as the standard deviation of normal-to-normal (R-R) intervals in milliseconds.

^c^ Described as percentage achieved of the estimated maximum HR based on age.

**Table 3:** Overview of statistical results from MANOVA analyses comparing scenario type, weapon rate of fire, and the interaction for analyses conducted using both behavioral data and physiological data.

	Behavioral analyses	Physiological analyses
Scenario type	Wilks’ Λ = 0.21, *η*_p_^2^ = .79**	Wilks’Λ = 0.74, *η*_p_^2^ = .14*
Weapon rate of fire	Wilks’ Λ = 0.17, *η*_p_^2^ = .83**	n.s.
Interaction	n.s.	n.s.

Notably, only 2 scenario types (shoot, fluid hostility) were used in the behavioral analyses MANOVA because all data associated with shots fired would be errors in don't-shoot scenarios. Analyses on those behavioral errors are presented separately in the Results section. n.s., denotes a non-significant result.

* denotes *p *< .05.

** denotes *p *< .001.

The multivariate effect was significantly different between the scenario types, Λ = 0.74, *F*(4, 66) = 2.67, *p *= .04, *η*_p_^2^ = .14. Univariate analyses indicated a significant main effect of scenario for HRV, *F*(2, 34) = 3.32, *p* = .05, *η*_p_^2^ = .16, but not for peak HR, *F*(2, 34) = 2.17, *p* = .13, *η*_p_^2^ = .11. After Bonferroni correction, the difference remained significant for HRV between the don't-shoot scenario and shoot scenario (lower HRV in the don't-shoot scenario; mean difference = 2.92 ms, *SE* = 1.12 ms, *p *= .05), although the difference between the don't-shoot scenario and fluid hostility scenario was not significant (mean difference = 3.51 ms, *SE* = 1.79 ms, *p *= .19). These differences reflect a general difference in HRV for scenarios where participants were supposed to fire their weapon vs. scenarios where they were intended to withhold fire, with the lower HRV associated with scenarios where someone should withhold fire (mean difference = 3.21 ms, *SE* = 1.43 ms, *p* = .03, *d* = 0.46). The shoot scenario and fluid hostility scenario were not significantly different (*p *> .05).

All other multivariate effects, main effects, and interactions were non-significant for both the HRV and peak HR dependent variables.

### Predicting shooting errors: unintended casualties

A logistic regression was conducted on the binary dependent variable of whether a false alarm occurred during the scenario. There were seven predictor variables: total shots fired during the scenario, number of non-hostiles in the scene, weapon rate of fire (categorical, SEMI or AUTO), number of lethal rounds fired in the scenario, and the physiological measures of HRV, %HR, and age. These combined variables predicted the likelihood of firing upon a non-hostile target, χ^2^(7) = 101.84, *p* < .001, Nagelkerke *R*^2^ = .43, with an overall correct classification rate of 82.49% (43.24% sensitivity, 95.52% specificity).

Five variables were significant predictors. Number of non-hostiles present increased the likelihood of an unintended casualty by nearly three times per additional non-hostile present [Wald χ^2 ^= 27.02, *p *< .001; odds ratio (OR): 2.85, 95% CI (1.92, 4.23)]. Number of lethal rounds fired decreased the likelihood of unintended casualties by more than half per lethal round fired [Wald *χ*^2 ^= 15.79, *p *< .001; OR: 0.38, 95% CI (0.24, 0.61)]. For every millisecond decrease in HRV, there was a 2% increase in the likelihood of inflicting an unintended casualty [Wald *χ*^2 ^= 6.38, *p *= .01; OR: 1.02, 95% CI (1.01, 1.04)]. For every percentage point increase in %HR, there was a 7% increase in the likelihood of inflicting an unintended casualty (Wald *χ*^2 ^= 5.49, *p *= .02; OR: 1.07, 95% CI [1.01, 1.12]. For every year of age, there was a 9% decrease in the likelihood of inflicting an unintended casualty (Wald *χ*^2 ^= 6.94, *p *< .01; OR: 0.91, 95% CI [0.84, 0.98]. The remaining predictors (shots fired, weapon rate of fire) were non-significant (*p *> .78). See Table 4 for common predictors.

**Table 4:** Common predictors between the linear regression model to predict lethal rounds fired and logistic regression model to predict unintended casualties inflicted.

Predictor variable	Lethal rounds	Unintended casualties
	Linear regression (β weights)	Logistic regression (OR)
Shots fired	.221	n.s.
Number of non-hostiles	−.210	2.85
Weapon rate of fire	.151	n.s.
Heart rate variability	−.132	1.02

n.s., non-significant predictor; OR, odds ratio.

### Predicting performance: lethal rounds fired

A linear regression was conducted with the dependent variable of lethal rounds fired. There were seven predictor variables: shots fired, first shot RT, number of non-hostiles in the scene, weapon rate of fire (categorical, SEMI or AUTO), and physiological measures (HRV, %HR, and age). These variables were able to significantly predict the number of lethal rounds fired, adj. *R*^2 ^= .13, *F*(7, 374) = 8.96, *p *< .001. Four predictors were significant in the model, including number of shots fired, number of non-hostiles present, weapon rate of fire, and HRV. An increased number of shots fired was more likely to result in a lethal shot fired (*β* = .221, *t* = 4.04, *p *< .001, *sr^2^* = .04), which was the strongest predictor in the model. The number of non-hostiles predicted the number of lethal rounds fired, with more lethal rounds fired when there were fewer non-hostiles (*β* = −.210, *t* = 3.95, *p *< .001, *sr^2^* = .04). Weapon rate of fire predicted the number of lethal rounds fired (*β* = .151, *t* = 2.86, *p *< .01, *sr^2^* = .02). AUTO rate of fire was more likely to produce lethal hits, a result related to the number of shots fired. Lastly, HRV predicted the number of lethal rounds fired (*β* = −.132, *t* = 2.24, *p *= .03, *sr^2^* = .01), with data suggesting that individuals with lower HRV had an increased likelihood of inflicting lethal rounds. The remaining predictors (first shot RT, %HR, age) were all non-significant (*p* > 0.05).

### Qualitative notes from physiological data collection in shooting simulations

The foremost concern involves accuracy of the physiological measurement given the theoretical issues associated with ultra-short HRV and physiological wearables, respectively. Scenarios utilized here averaged 43.61 s in duration [standard error (SE) = 0.39 s]. Although individual scenarios can vary greatly based on the training intent and specific organization, this evidence confirms that existing shooting simulations can fall well below recommended duration for physiological measurement. The concern then becomes the amount of physiological information that could be collected. In these shooting scenarios, there was an average heart rate of 95.47 beats per minute (bpm; SE = 3.23 bpm), which creates, on average, only 69.39 heartbeats during a single scenario (SE = 2.42 heartbeats). The physiological monitors used here calculate HRV based on the last 300 beats (41), which means that the actual physiological information related to heart rate is a product of both the scenario itself and the remaining baseline period.

The practical lesson here differs for physiologists and use-of-force practitioners. Physiologists will note that the restricted timeframe has both theoretical and methodological implications for measurement. In particular, the timeframe potentially restricts analyses to ultra-short HRV windows. However, the context also heightens the theoretical importance of HRV as a predictor since the actual shooting portions of these scenarios last much less than even these abbreviated time frames—actual shooting may only last seconds, or less. Anticipatory HRV could be valuable to explore instead since HRV at the time of a shooting error remains downstream from the stress response preceding it. Therefore, the contrast between anticipatory stress prior to the scenario and acute stress following the shooting scenario might be a valuable metric to capture in future measurement during shooting simulations.

For practitioners seeking direct implementation of physiological measurement into shooting simulations, the implications involve pacing. A false presumption would be that the short timeframe means the current scenario contains HRV information from a previous scenario, but this possibility does not happen automatically. Shooting simulators typically require the practitioner to transition between scenarios, and so there could be at least a minute between scenarios plus time to address any weapon issues (e.g., refilling the magazine or replacing used magazines). Additionally, a training instructor will likely spend several minutes debriefing the participant on performance from the last scenario. This time, if applied correctly, could be used to prevent overlap between scenarios without requiring a full 5-minute inactive baseline to distinguish performance. Anticipatory and recovery metrics could also be important variables to consider here.

Granted, these variables become relevant only when trying to link performance to particular events. The trainer assumption is likely to be, and not inaccurately, that current physiological metrics have been substantially influenced by the previous scenario. This assumption may not fully capture the accumulation of stress throughout a training session as participants become stressed or fatigued by cumulative events. Except for highly experienced participants, merely the requirement of holding a rifle for this long period requires frequent rest. Trigger finger fatigue is also a real possibility as most people are unaccustomed to firing a weapon with recoil several hundred times (for this study, mean shots fired = 156.11 rounds, SE = 16.08 rounds). The weapon itself can then have implications for fatigue given its weight and relative rate of fire given the recoil implications and variable trigger pull rate between weapons. Movement is another critical factor to consider since the change in HR induced by physical stress could significantly confound any of the HRV measurements. However, many shooting simulators require largely static posture without the ability for significant physical movement, and so the stressor remains largely cognitive. This caveat depends greatly upon the shooting simulator though as some setups do permit greater physical movement during the scenarios.

## Discussion

Recent efforts have noted the advantage of integrating physiological monitoring into applications for military, law enforcement, and security personnel (30, 44). In particular, there is interest in utilizing physiological monitoring within shooting simulators for training and evaluation purposes. While similar assessments during physical performance are well-established, physiological monitoring in shooting simulations may not be a simple integration for several reasons. Theoretical problems arise given the challenges associated with ultra-short HRV measurements (33, 35, 37). Practical problems arise because existing libraries of training scenarios were designed to emulate realism in shoot scenarios that impose practical stress. Unfortunately, these caveats often mean that the scenarios are too short in duration for any typical HRV measurement. Another challenge involves identifying what information, if any, might be useful to extract from physiological monitoring for applied purposes. The combination suggests that integrating off-the-shelf commercial wearable technology into use-of-force scenario training could provide some misleading results unless the scenarios were to be augmented or additional controls were introduced. As such, the current study sought to integrate physiological monitoring into existing use-of-force training scenarios to explore the potential pitfalls of direct implementation of wearable technology in shooting simulations.

Overall, lower HRV was observed in scenarios where participants did not fire a shot compared to scenarios where participants did have to fire. If future work can replicate this finding, it would indicate that participants are more stressed by scenarios where they need to withhold a shot rather than scenarios where they actually engage a target—at least, during simulations. The likelihood of inflicting an unintended casualty was also related to increased %HR and decreased HRV, indicative of elevated sympathetic drive. As stress increases and autonomic inputs correspondingly increase, HR accelerates to increase blood flow for muscles and vital organs, priming the system to fight or flee (45). It is well known that there is a fine balance between optimal stress for performance and too much stress, which may lead to failure [cf. (46, 47)], characterized here as shooting an unarmed person or non-hostile. Elevated HR has also been associated with decreases in decision-making capabilities under duress (48, 49). This interaction creates a complex system of cognitive and physiological feedback, with an optimal window for human performance that benefits from some stress and fails when overloaded.

This exploratory implementation created several lessons learned. First, many existing scenarios may be too short for viable physiological measurement. Ultra-short HRV may be able to capture some relevant information, but there is potential for scenario cross-over if the training simulation does not plan enough time between scenarios. The practical implementation would be to have a controlled debriefing with continuous physiological monitoring to capture recovery from the event. This would be informative to identify individuals where the stress may be compounding and thus put an individual in a hyper-aroused state. Monitoring recovery will enable mitigation strategies [breathing, mindfulness] by instructors, especially for novice individuals. Ultimately, the easiest implementation would be omnibus physiological monitoring rather than scenario-based physical monitoring. The difference involves whether performance is linked to specific scenarios. Omnibus monitoring could provide insight into stress and immersion as well as anticipation of a stressful event, execution of the event, and recovery—all analogues to a sport scenario. Longitudinal monitoring over time will enable determination of an individual is adaptation to the stressor [inoculation] and provide opportunity for intervention [mental skills training] to manage the stress load and decrease risk of making a fatal error [cf. (50)].

Behaviorally, participants fired 86% more shots per scenario with an automatic weapon, which was coupled to an increase in both lethal (71%) and non-lethal rounds (56%) fired during automatic weapon scenarios. Contrary to previous work (38), false alarms were not increased with an automatic weapon compared to a semi-automatic weapon. This discrepancy is likely an effect driven by differences in shooting simulators. Previous work utilized a low-fidelity gaming platform with a higher rate of intermixed hostile and non-hostile actors, whereas hostile and non-hostile targets did not overlap physically on screen as much in the military-grade simulations. This ratio of hostiles to non-hostiles within a scenario is a powerful factor in evoking shooting errors [cf. (51)], and in the regression analyses, the number of non-hostiles was the primary driver of unintended casualties. Combined with the increased number of shots fired while using the automatic weapon, these factors seem to explain why the previous evidence showed a difference due to rate of fire and the current findings demonstrated no difference. However, it is worth noting that while the scenarios are more realistic in general, the simulator differs in one major capacity: recoil. The ISMT used in the current study provided recoil during fire, which may have impacted how each participant engaged their targets. Collectively, the differences in data collection systems may have resulted in variations to weapon handling as well as hostile target identification. Another important difference involves accuracy when considering real-world implications. Automatic weapons in simulators lack the recoil that significantly impairs accuracy with an automatic weapon. While real-world automatic fire would similarly involve more shots fired, as observed here, accuracy differences should be anticipated when comparing real-world weapons with automatic vs. semi-automatic rate of fire.

Arguably, the overlap between scenarios becomes the most important consideration—both for physiological monitoring and training. Most shooting simulations are not designed to integrate physiological measurement, and each organization will use their shooting simulators differently for training purposes. The inherent variability in application and procedure makes any plug-and-play applications of physiological monitoring difficult. From a training perspective, data interpretations could be biased as observed anxiety could bleed over from one scenario to the next. This issue limits how much an instructor should interpret anxiety from a particular scenario, although there could be value in examining elevated anxiety across a variety of scenarios. Practically, from a physiological measurement perspective, the average scenario is too short to prevent HRV calculations as a rolling average from blending performance across multiple scenarios. It would be possible to use briefing and debriefing periods between scenarios to buffer some of this issue, yet the problem remains pervasive. Future solutions would need to develop specific scenarios around the physiological measurement to better depict the data collected as attributable to a current shooting simulation. If employing existing shooting libraries as the basis for physiological measurement, then any implementation must be prepared to address the substantial bleed through between scenarios. Further interesting applications could explore how the success or failure of previous trials influences subsequent performance, both for physiological monitoring and practical training applications. Most studies and training procedures do not account for inter-trial effects, making the idea a fruitful area for future research.

An important consideration involves the use of SDNN to measure HRV rather than root mean square of successive differences between normal heartbeats (RMSSD). The reason for selecting SDNN over RMSSD was a practical one—the Zephyr system reported HRV values in SDNN. As such, equipment was the reason to select SDNN over RMSSD as the Zephyr system was available for this exploratory effort, yet RMSSD may offer a better metric for ultra-short HRV than SDNN (52). This possibility has several implications for the current findings. Most notably, the odds ratios reported here for unintended casualties were relatively modest for HRV measures. This outcome could be attributed to the importance of shooting behaviors in the scenarios as a better indicator of stress reaction than the physiological measure, or the finding could be due to the reduced sensitivity of the SDNN measure in the ultra-short HRV window. Either possibility could explain the observed odds ratios. Other evidence suggests that SDNN could still capture HRV accurately in a 30 s window (37), albeit this limitation too warrants consideration as these shooting scenarios lasted longer than 30 s while the key event (i.e., having to engage a target) might not have occurred until very late in the scenario. This timing sequence could therefore limit any evidence HRV could have if measured as an average across the scenario. Additional research would be necessary to dissociate the roles of device and behavior in better capturing individual reactions to shooting scenarios in simulators.

Future directions could lead physiological monitoring in use-of-force training down multiple pathways. Notably, force-on-force training scenarios with non-lethal ammunition may elicit physiological response through pain sensations (53–55). These simulated engagements are more realistic than shooting simulations as they involve engaging live opponents rather than video simulations. Even so, those scenarios are necessarily contrived with protective gear and limited engagement potential. Another concern involves the non-lethal weapons, which may not be as accurate or precise beyond a certain distance due to the training rounds used. The combination of force-on-force training and non-lethal weapons suggests that realistic training methods may appropriately simulate anxiety without adequately replicating other cognitive factors related to performance. Physiological assessments here also focused on the overall RT and decision making during the scenario vs. isolation of an exact moment such as trigger pull time (56) or variations in HR through a prolonged target engagement (57). These differences have significant implications for measuring physiological factors in existing training simulations.

In conclusion, advancements in technology have enabled marksmanship training to occur in safe environments with unpredictable scenarios. These dynamic training environments not only serve to improve decision making under stress, but also aim to decrease fatality in kinetic environments in which our military and law enforcement individuals are exposed. As such, there is an emerging appetite to integrate psychophysiological elements into behavioral shooting assessments (58). This study served as an exploratory evaluation of direct integration of physiological assessments into existing training libraries conducted in tandem with a behavioral assessment of weapon rate of fire. Outcomes demonstrated that the semi-automatic fire and automatic fire resulted in similar outcomes. The data presented herein demonstrate the importance of simulator design and scenarios as potential confounders for behavior. Moreover, if physiological metrics are to be collected, it may not be optimal to simply integrate physiological measurement into existing training libraries. Trainers and physiologists should work together to develop scenarios that optimally capture physiological variables as indicators of stress management.

## Data Availability

The datasets presented in this article are not readily available because data remain property of the U.S. government. Requests for access should follow appropriate procedures to request government data. Requests to access the datasets should be directed to karen.r.kelly8.civ@health.mil.
